# Endoscopic retrograde appendicitis therapy for giant periappendiceal abscess with intestinal obstruction

**DOI:** 10.1055/a-2173-7756

**Published:** 2023-10-06

**Authors:** Qianlong Li, Tianyu Liu, Aiying Li, Jing Liu, Biao Jiang, Bo Yang

**Affiliations:** Fourth Department, Digestive Disease Center, Suining Central Hospital, Sichuan, China

In recent years, endoscopic retrograde appendicitis therapy (ERAT) has been widely used in the treatment of acute uncomplex appendicitis 1 . However, the treatment of a periappendiceal abscess has always been a challenge. Early surgery may cause serious complications such as total peritonitis and postoperative anastomotic leakage 2 . We report a case of acute appendicitis with a giant periappendiceal abscess and intestinal obstruction. Through ERAT, full endoscopic drainage was achieved and the intestinal obstruction was quickly resolved.

A 34-year-old woman presented with abdominal pain, nausea, vomiting, and no anal exhaust defecation for 2 days. Computed tomography (CT) showed acute appendicitis with a giant periappendiceal abscess and intestinal obstruction ( Fig. 1 ). After informed consent was given, an ERAT procedure was performed ( Video 1 ) with the patient under general anesthesia. The intestine was prepared through colon dialysis. Colonoscopy showed significant protrusion of the mucosa around the appendix. The appendiceal cavity was successfully incised using a duodenal papillary incision knife ( Fig. 2 ). Then a guidewire was delivered to the giant periappendiceal abscess, which was clearly visualized by iohexol radiography ( Fig. 3 ). After that, a biliary plastic stent was successfully implanted. A large amount of pus flowed out of the stent ( Fig. 4 ). The procedure was performed successfully without any adverse events. Postoperative abdominal X-ray indicated no signs of intestinal obstruction. The patient recovered well.

**Fig. 1:**
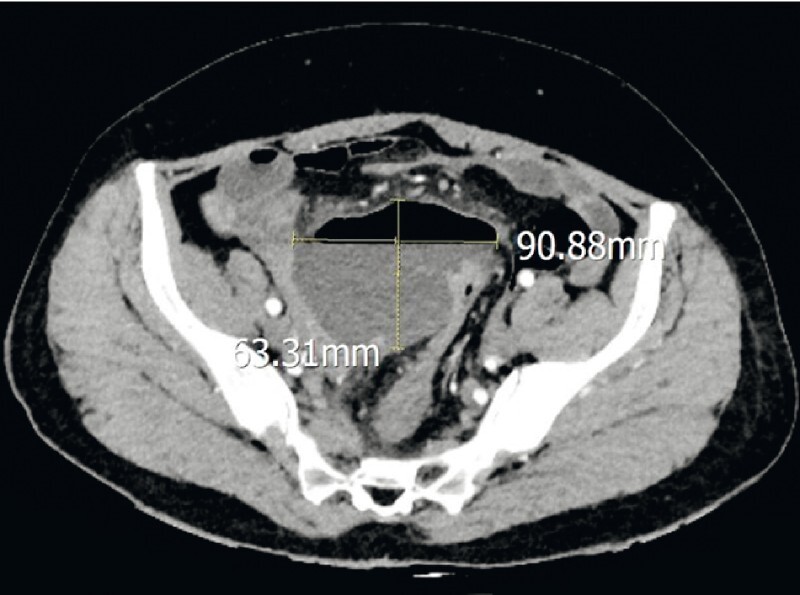
Before endoscopic retrograde appendicitis therapy, computed tomography (CT) showed acute appendicitis with a giant periappendiceal abscess (9.0 cm × 6.3 cm) and intestinal obstruction.

**Video 1** Endoscopic retrograde appendicitis therapy for continuous drainage of giant periappendiceal abscess and the process of removing the stent under colonoscopy.


**Fig. 2:**
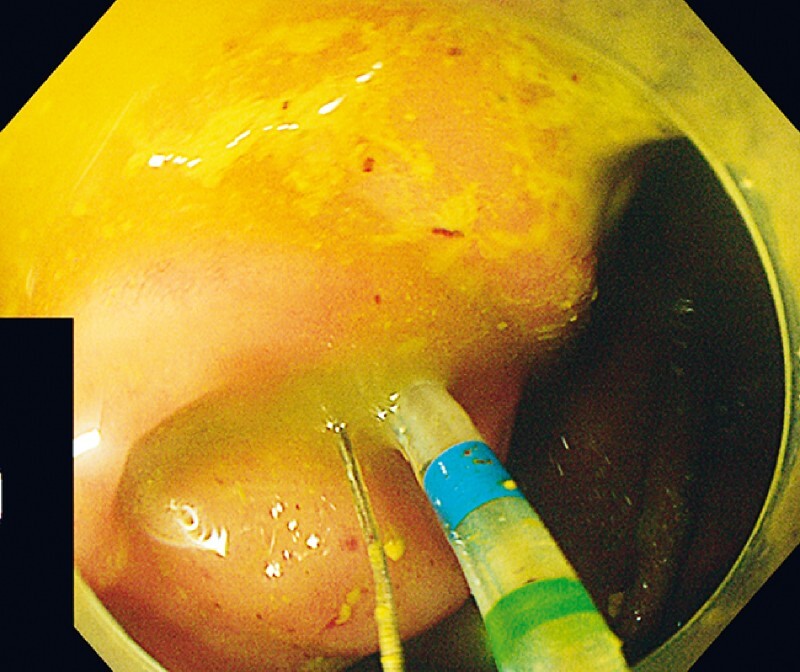
Colonoscopy showed protrusion of the mucosa around the appendix. The duodenal papillary incision was used for successful insertion into the appendix cavity.

**Fig. 3:**
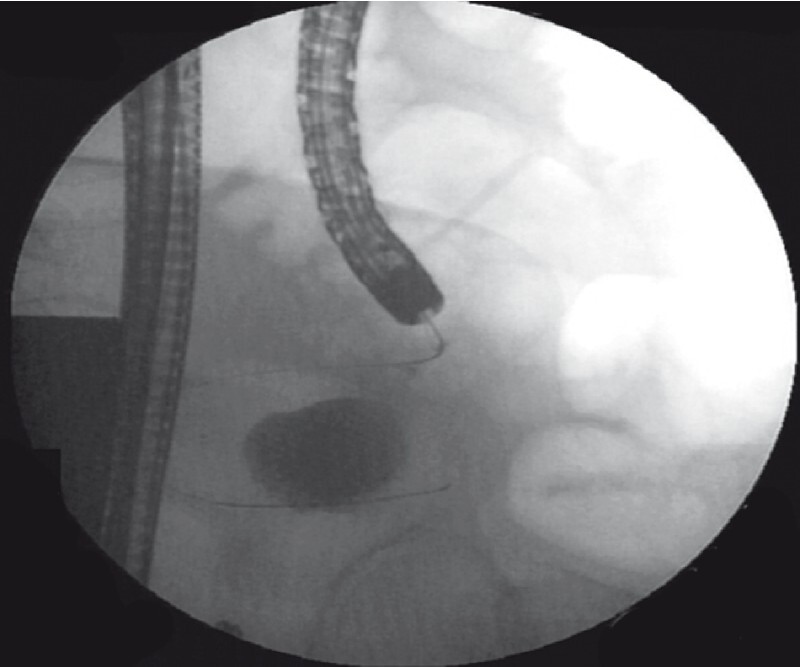
The guidewire was delivered to the appendix pus cavity, and then iohexol radiography clearly showed the giant periappendiceal abscess.

**Fig. 4:**
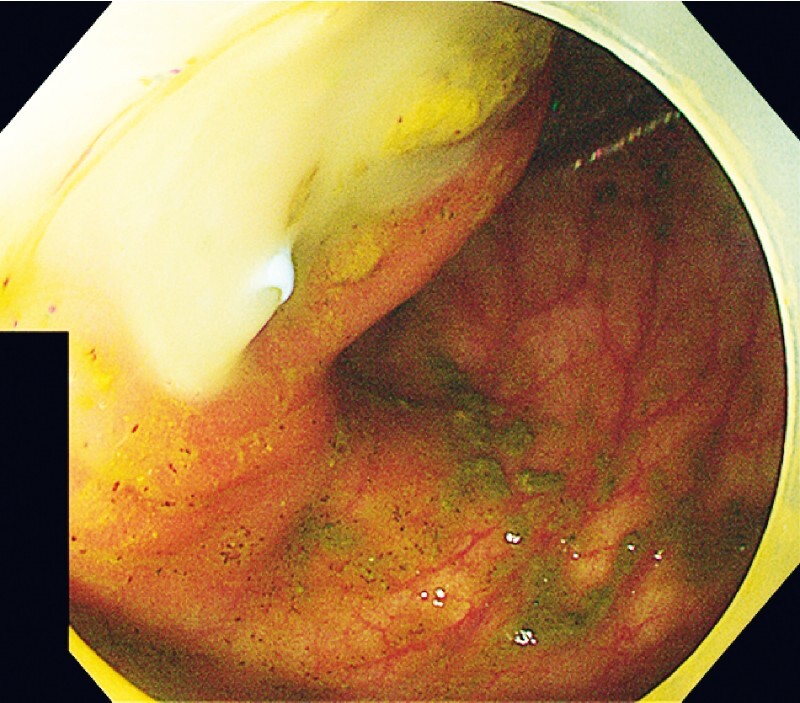
After a plastic biliary stent was implanted into the appendix pus cavity, a large amount of pus flowed out of the stent.

Follow-up CT after 2 months showed that the periappendiceal abscess had fully disappeared ( Fig. 5 ). The stent was removed under colonoscopy ( Video 1 ). ERAT may be a safe and effective method for adequate drainage of a periappendiceal abscess in the early stage. Further clinical studies with larger samples and long-term follow-up are needed to evaluate this.

**Fig. 5:**
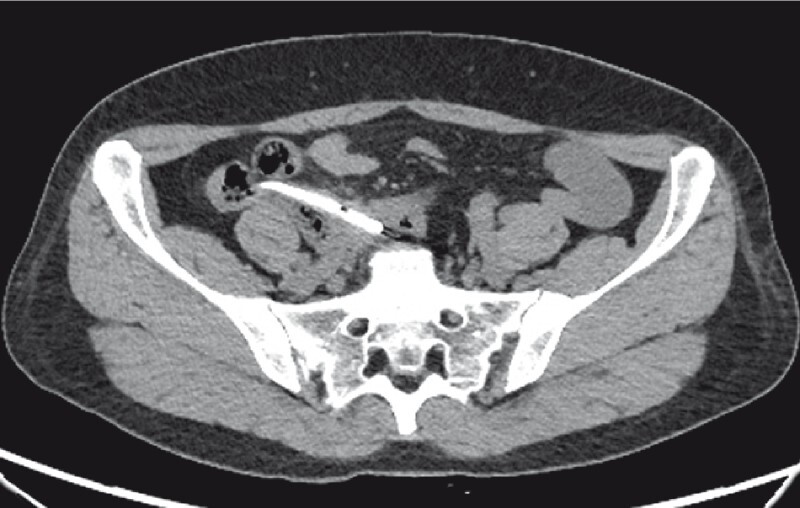
Follow-up CT after 2 months showed that the periappendiceal abscess had fully disappeared and the stent was in place.

Endoscopy_UCTN_Code_CCL_1AD_2AG

## Notes

Endoscopy E-Videos

https://eref.thieme.de/e-videos
*E-Videos* is an open access online section of the journal *Endoscopy* , reporting on interesting cases and new techniques in gastroenterological endoscopy. All papers include a high-quality video and are published with a Creative Commons CC-BY license. Endoscopy E-Videos qualify for HINARI discounts and waivers and eligibility is automatically checked during the submission process. We grant 100% waivers to articles whose corresponding authors are based in Group A countries and 50% waivers to those who are based in Group B countries as classified by Research4Life (see: https://www.research4life.org/access/eligibility/ ). This section has its own submission website at https://mc.manuscriptcentral.com/e-videos
